# Reflections on the RSA guidelines

**DOI:** 10.1080/17453674.2020.1763568

**Published:** 2020-05-12

**Authors:** Bart G Pijls

**Affiliations:** Department of Orthopaedics, Leiden University Medical Center, Leiden, The Netherlands

Roentgen stereogrammetric analysis/radiostereometric analysis (RSA) is an internationally accepted and validated surrogate marker for long-term primary joint replacement outcome in terms of aseptic loosening and its positive effect on patient safety even echoes through in national joint registries (Ryd 1986, Nelissen et al. 2011, Kärrholm 2012). The history of RSA dates back to the time when X-rays were discovered, when Davidson and Hedley determined the 3-D position of a pin that was radiographed on the same radiograph by two separate x-ray sources (Davidson and Hedley 1897). Modern RSA dawned in the 1970s and has first been reported by Göran Selvik in his thesis (Selvik 1989). In 2005 a landmark paper in the field of RSA was published by an international group of RSA experts: “Guidelines for standardization of radiostereometry (RSA) of implants”, also referred to as “The RSA guidelines” (Valstar et al. 2005). Presently, this paper counts over 400 citations in google scholar (accessed 19-02-2020). Although this number of citations is impressive, the measurable impact on the reporting quality of RSA studies is even more impressive. Since its publication, the reporting quality of RSA studies has greatly improved; Madanat et al. (2014) have shown that the proportion of RSA studies with high reporting quality increased almost 3-fold in the period 2006-2011 compared to the period before the RSA guidelines were published. The RSA guidelines also formed the foundation for the ISO standard ISO 16087:2013: Implants for surgery — Roentgen stereophotogrammetric analysis for the assessment of migration of orthopaedic implants (ISO 16087:2013). This ISO standard facilitated further optimization and professionalization of RSA research. Additionally, some of the recommendations in the RSA guidelines on e.g. the use of signed values, the rationale for using (or not using) Maximal Total Point Motion (MTPM) and the timing of the first postoperative examinations are still very relevant today.

The importance of adequate reporting is being recognized across all fields of health care research. Reporting guidelines are considered vital for achieving and maintaining high standards in reporting healthcare research and avoiding waste in the production and reporting of research (Altman and Simera 2016). As such, reporting guidelines specify the minimum information that is needed for a reader to get a clear and complete picture of what was done, what was found and what the results mean, so the study can be completely understood, replicated, appraised and the results be interpreted in the correct context (Altman and Simera 2016). It is important to realize that although high reporting quality is required to judge the methodological quality of a study, high reporting quality does not mean the methodological quality is high as well. Perfectly reported studies may be poorly designed and poorly executed and vice versa. For example, a lost to follow-up of 50% in a study indicates poor methodological quality. However, when this fact is clearly reported, indicating high reporting quality, it allows the readers to adequately appraise the methodological study quality. Hence, high reporting quality is a prerequisite to judge the methodological quality of a study.

Regarding RSA studies, the reporting quality has improved, but further improvement is possible and necessary (Madanat et al. 2014). In a recent review on migration in total knee replacements it became apparent that RSA studies could benefit from further improvement in the reporting of especially the migration results and precision as determined by double examinations: only 19 of 53 included studies reported precision as determined by original double examinations (Pijls et al. 2018). Clinicians, researchers, clinical guide line developers, systematic reviewers and patients would greatly benefit from standardized and complete reporting of prosthetic migration e.g. the mean migration, the number of RSA examinations for each follow-up and detailed description of the type of prosthesis and fixation method.

To further improve reporting and transparency it should become standard practice to register RSA studies (including case series and cohorts) in a trial registry e.g. at clinicaltrials.gov before start of the study and data-analysis, as is common practice for randomized controlled trials. Although study registration is not yet compulsory for RSA cohorts and case-series, proper study registration of such studies would ensure assessment of publication bias especially when the migration of new prostheses exceed the unacceptable thresholds.

The RSA guidelines have greatly enhanced reporting of RSA studies and have paved the way for further improvements especially regarding the reporting of migration results and study registration in trial registries.

Bart G Pijls Department of Orthopaedics, Leiden University Medical Center, Leiden, The Netherlands *E-mail:*b.g.c.w.pijls@lumc.nl

## References

[CIT0001] Altman D G, Simera I. A history of the evolution of guidelines for reporting medical research: the long road to the EQUATOR Network. J R Soc Med 2016; 109(2): 67–77. 26880653 10.1177/0141076815625599 PMC4793768

[CIT0002] Davidson J M, Hedley W S. A method of precise localisation and measurement by means of roentgen rays. Lancet 1897; 65(16): 1001.

[CIT0003] ISO 16087:2013(E). Implants for surgery: roentgen stereophotogrammetric analysis for the assessment of migration of orthopaedic implants. Geneva: International Organization for Standardization; 2013.

[CIT0004] Kärrholm J. Radiostereometric analysis of early implant migration – a valuable tool to ensure proper introduction of new implants. Acta Orthop 2012; 83(6): 551–2. 23126576 10.3109/17453674.2012.745352 PMC3555445

[CIT0005] Madanat R, Mäkinen T J, Aro H T, Bragdon C, Malchau H. Adherence of hip and knee arthroplasty studies to RSA standardization guidelines. A systematic review. Acta Orthop 2014; 85(5): 447–55. 24954489 10.3109/17453674.2014.934187 PMC4164860

[CIT0006] Nelissen R G, Pijls B G, Kärrholm J, Malchau H, Nieuwenhuijse M J, Valstar E R. RSA and registries: the quest for phased introduction of new implants. J Bone Joint Surg Am 2011; 93(Suppl 3): 62–5. 10.2106/JBJS.K.00907 22262426

[CIT0007] Pijls B G, Plevier J W, Nelissen R G. RSA migration of total knee replacements. Acta Orthop 2018; 89(3): 320–28. 29508661 10.1080/17453674.2018.1443635 PMC6055769

[CIT0008] Ryd L. Micromotion in knee arthroplasty. A roentgen stereophotogrammetric analysis of tibial component fixation. Acta Orthop; 1986; Suppl 220: 1–80. 3461667

[CIT0009] Selvik G. Roentgen stereophotogrammetry. A method for the study of the kinematics of the skeletal system. Acta Orthop 1989; Suppl 232: 1–51. 2686344

[CIT0010] Valstar E R, Gill R, Ryd L, Flivik G, Borlin N, Kärrholm J. Guidelines for standardization of radiostereometry (RSA) of implants. Acta Orthop 2005; 76(4): 563–72. 16195075 10.1080/17453670510041574

